# Rugged, low-cost, and lightweight rosette water sampler for ocean profiling and mooring deployment

**DOI:** 10.1016/j.ohx.2025.e00661

**Published:** 2025-05-31

**Authors:** Ebbe Poulsen, Søren Rysgaard, Peter Melvad, Claus Melvad

**Affiliations:** a Arctic Research Centre, Department of Biology, Aarhus University, Ole Worms Allé 1, DK-8000 Aarhus C, Denmark; b Department of Mechanical and Production Engineering, Aarhus University, Katrinebjergvej 89 G-F, DK-8200 Aarhus N, Denmark

**Keywords:** Rosette water sampler, Ocean profiling, Depth controlled, Low-cost, Lightweight, Efficient

## Abstract

Oceanographers studying climate change, marine ecosystems, and water mass distribution rely heavily on seawater measurements from various depths and locations. A critical tool for obtaining these measurements is the rosette water sampler, which collects and isolates seawater at multiple specified depths for later analysis. However, most commercially available samplers are designed for large sample volumes (>500 mL), requiring heavy-duty lifting equipment typically found only on larger research vessels.

This creates unnecessary costs and operational challenges, especially in coastal areas, when large sample volumes are not needed. In response, we present a small, lightweight, and rugged rosette water sampler specifically developed for smaller sample volumes and efficient deployment from small boats without the need for lifting equipment. Weighing 16 kg, the sampler can collect 13 samples of 20 mL each in a single profiling cast, with a maximum depth of 250 m. The instrument has been successfully tested in Northeast Greenland and along the East Greenland coast during three field campaigns between 2021 and 2023. Further development could lead to additional weight reductions and improved ease of use, enhancing its practicality for broader applications.

## Graphical abstract

**Figure:**
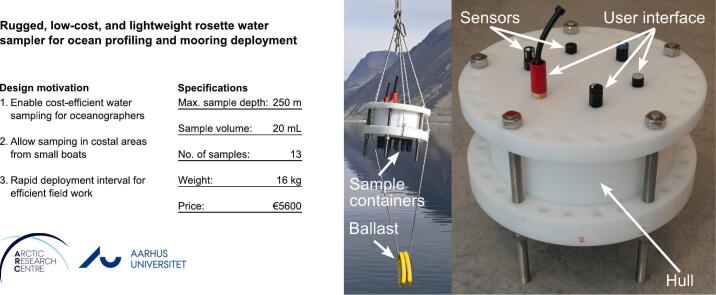

**Table:** 

**Specifications table**	
Hardware name	Arctic Research Centre Rosette Water Sampler (ARC-RWS)
Subject area	Environmental, planetary and agricultural sciences
Hardware type	Field measurements and sensors
Closest commercial analog	Sea-Bird Scientific SBE 32 Carousel Water Sampler
Open source license	CC BY 4.0
Cost of hardware	€ 5600
Source file repository	https://doi.org/10.17632/b3jms757m9.2

## 1 Hardware in context

**Fig. 1:**
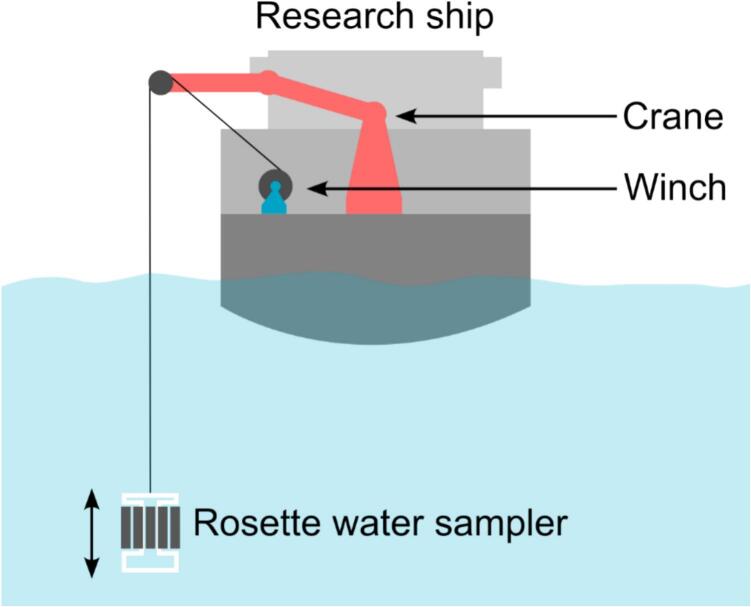
Typical principle of operating a rosette water sampler from a research vessel.

Water samplers are an important tool in oceanography because they allow scientists to collect and analyze seawater from different depths and locations, providing valuable data on the ocean’s physical, chemical and biological properties. These samples help monitor temperature, salinity, nutrient levels, alkalinity, isotopes and biological content like plankton. By studying water samples, oceanographers can better understand the distribution of water masses, marine ecosystems, track climate change, and assess the health of ocean environments, contributing to global environmental monitoring and conservation efforts. Collecting water samples in different depths is commonly carried out using casts with rosette water samplers that capture and seal samples on pressure sensor input, timers, or weights (messengers) sent down along the winch wire [1]. These use multiple individual samplers mounted in a circular pattern, with a central sensing and actuating unit. Fig. 1 illustrates how rosette water samplers are commonly deployed from research vessels.

Rosette water samplers are a proven and reliable technology often using the Niskin bottle, but systems commercially available today typically focus on sample volumes larger than 500 mL [[2], [3], [4], [5], [6]], generally increasing the size and weight of the sampler. When the needed sample volume is significantly lower (<50 mL) this creates an unnecessary requirement for heavy duty lifting equipment mainly found on larger research vessels, resulting in a higher operating cost and operational restrictions in coastal areas. The idea of a small, light, and fast acting water sampler for efficient sampling of small sample volumes from small boats was formed. To materialize this idea, the requirements in Table 1 was considered essential by the authors based on extensive field experience using existing systems.

**Table 1:** Main requirements for the ARC-RWS.

Requirement	Value/specification	ARC-RWS	Justification
Sampling depth	Min. 250 m	250 m	Must not be limited to shallow waters
Sampling volume	Min. 20 mL	20 mL	Analysis requirements
Dimensions	Max. 750x600x400 mm	350x350x300 mm	Logistics and handling
Weight	Max. 20 kg	16 kg	Logistics and handling, operation from small boats
Price	Max. €10.000	€5600	Competitive pricing making the instrument more attractive to a wider group of researchers
Number of samples	Min. 10	13	Operational efficiency and scientific value
Handling	2 pers.	1 pers.	Operation from small boats
Profiling time	Max. 15 min.	9 min.	Operational efficiency
Turn-around time	Max. 30 min.	10 min.	Operational efficiency
Operational temperature	−5 °C to 25 °C	−5 °C to 25 °C	Environmental
Pressure sensor accuracy	Max ± 150 mbar	200 mbar	Sampling precision

Smaller systems that utilize syringes or other smaller bottles for sampling do exist, but are generally slow acting, proving inefficient in a profiling setup [7,8] and thus not fulfilling the profiling time requirement in Table 1. Other systems with profiling time, size and weight within the requirements listed in Table 1 are available [9,10], but outside the price requirement (>€12.000). Low-cost open-source options do exist [11], but are limited to shallow waters, restricting their use. No system that enables efficient, low-cost and versatile sampling is currently available, which is why the Arctic Research Centre Rosette Water Sampler (ARC-RWS) was developed.

## 2 Hardware description

**Fig. 2:**
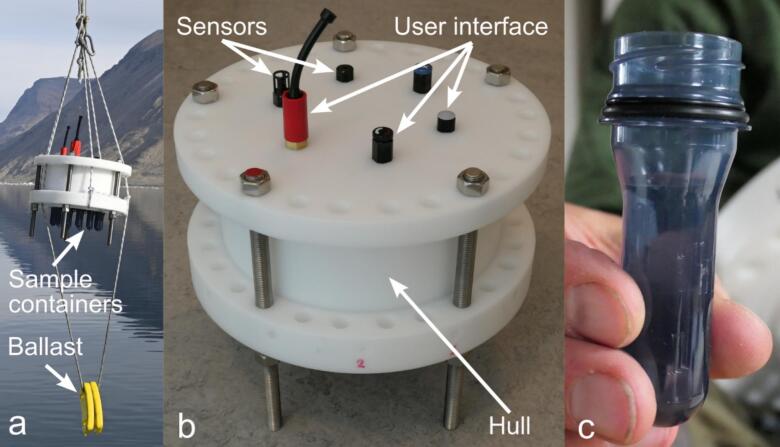
Overview of the ARC-RWS during deployment with ballast attached (a) close-up before deployment with no ballast attached (b) and closeup of a single sample container when removed from the instrument (c). Courtesy of Peter Bondo Christensen and the authors.

**Fig. 3:**
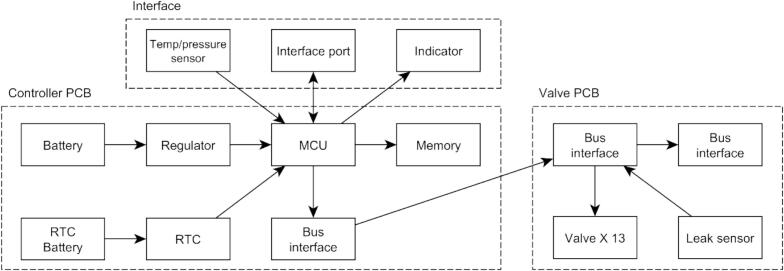
Diagram of the ARC-RWS system components.

**Fig. 4:**
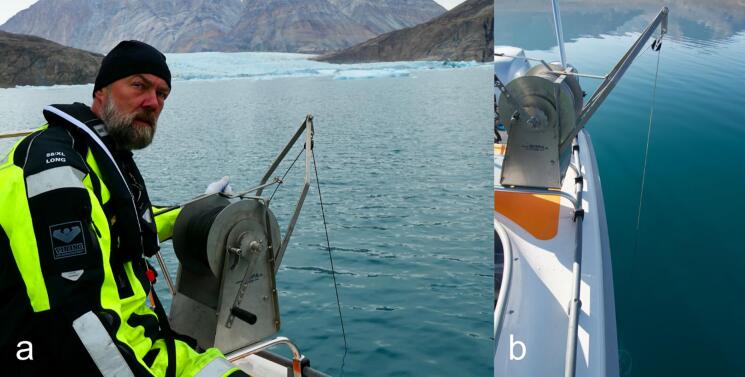
The ARC-RWS system during profiling from a small boat with manual winch (a) and immediately prior to resurfacing (b).

The ARC-RWS is a rugged, easy to use and compact rosette water sampler with a total weight of 16 kg, capable of collecting water samples at 13 user configurable depths. The design uses low-cost plastic sample containers that are easily removed and secured after sampling. The sampling is controlled by an integrated pressure sensor and sampling depths are recorded on the instrument along with temperature measurements. The instrument is limited to a maximum depth of 250 m. The ARC-RWS can easily be attached to a winch using rope though the external mounting holes. It is light and compact enough to be used down to the full rated depth on non-motorized winches (maximum winch pull is 50 N). The maximum dive speed can be controlled by varying the amount of ballast connected to the instrument. For an overview of the ARC-RWS see Fig. 2. A diagram of the complete system is included in Fig. 3. Images of the system in use are shown in Fig. 4. A video showing the deployment using a crane from a larger vessel is included in the design file repository (see section 3).

The sample containers used by the ARC-RWS are available in very wide range of sizes. The sample containers used currently have a sample volume of 20 mL, but larger samples are possible and only limited by a maximum outside diameter of 40 mm and the length of the threaded rods used as feet for the instrument. Installing longer threaded rods allows longer sample vials. Using larger sample containers can potentially increase the sample size to more than 50 mL.

The sampler uses a separate solenoid valve for each sample in a circular pattern to control the flow into the sample container. The valves are opened at the user specified depths during both down- and upcast and the water pressure is used to fill the sample. This allows for a low sample time resulting in a continuous and cost-effective cast.

The mechanics and electronics are designed to allow up to four units to be stacked together for a larger instrument of 52 samples that can be used to sample at regular intervals in a mooring configuration for up to a year. The focus of this manuscript is the single-unit version to keep the manuscript length short, but the reader is made aware of the possibility to extend the instrument.

As a consequence of designing the ARC-RWS with low-cost components in mind, the temperature and pressure sensors are not of comparable accuracy to most commercial conductivity, temperature, and depth (CTD) profilers used for oceanographic research. If higher accuracy is needed, an external CTD can be attached to the winch line. Several lightweight options exist that fit the ARC-RWS use case [12,13].

The key advantage of the ARC-RWS design is the ability to operate efficiently from small boats without lifting equipment and motorized winch systems. This allows sampling in coastal areas inaccessible to larger research vessels, as well as being highly cost efficient. Furthermore, the use of removeable sample containers significantly lowers the turn-around time between casts. The lower operating and purchase cost of the ARC-RWS, along with the ease of use, increases the potential of the system to be used in citizen science projects and for educational purposes. This also makes it a good fit for integration into larger observational programs, such as the Global Ocean Observing System (GOOS) [14]. In summation, benefits of the ARC-RWS design are:

- Operation from small marine vessels
  o Low operational cost
  o No crane needed for deployment
  o Can be used on a manual winch
- Low sampler turn-around time (sample extraction and preparation for next cast)
- Profiling and mooring capabilities
- Extremely easy to use
  o Suited for citizen science and educational projects
- Low-cost

The instrument consists of three subsystems: the hull, valve and sample containers, and control electronics. Each subsystem will be presented below.

### 2.1 Hull

**Fig. 5:**
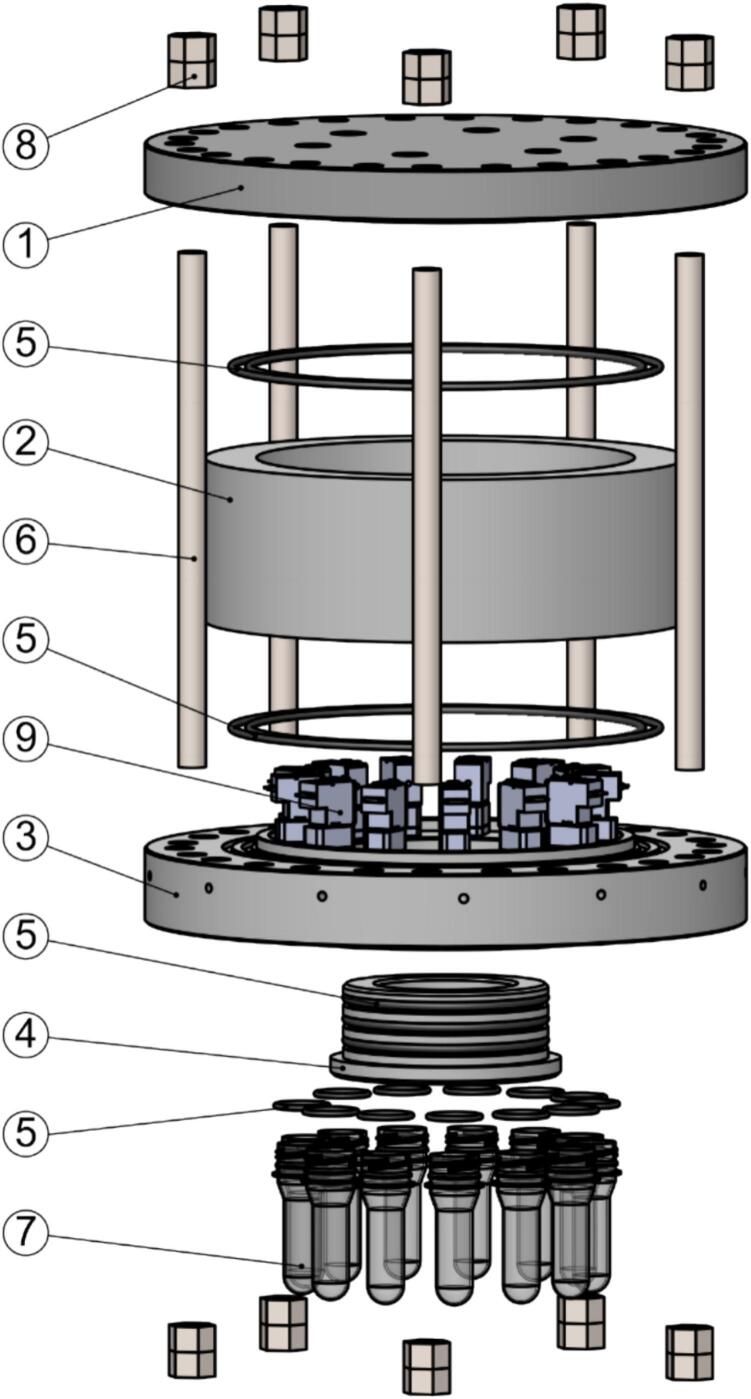
Exploded view of the main mechanical components of the ARC-RWS. (1) Top hull section, (2) hull cylinder, (3) valve manifold, (4) bottom endcap, (5) O-rings, (6) M16 threaded rods, (7) sample containers, (8) M16 nuts, and (9) valves.

Fig. 5 shows the main mechanical components of the ARC-RWS. The pressure hull consists of five machined components, the top hull (1), hull cylinder (2), valve manifold (3), bottom endcap (4), and sample container inserts (not shown). These are all made from polyoxymethylene (POM) plastic, allowing for a rugged, non-corrosive and very low maintenance system. The interface between each component is sealed by a double or triple O-ring seal.

The hull has been designed for depths of up to 250 m with a safety factor of 1.2, and this has been verified through field tests (see section 7. Validation and characterization).

The hull assembly is clamped together using stainless steel M16 threaded rods that also function as sturdy feet for the instrument.

The top hull section serves as the user interface for operating and configuring the instrument. The 13 sample containers and valves are installed at the bottom of the hull through the valve manifold. The valve manifold has a small opening for each valve, through which each sample container is filled when the valve is open.

### 2.2 Valve and sample container

**Fig. 6:**
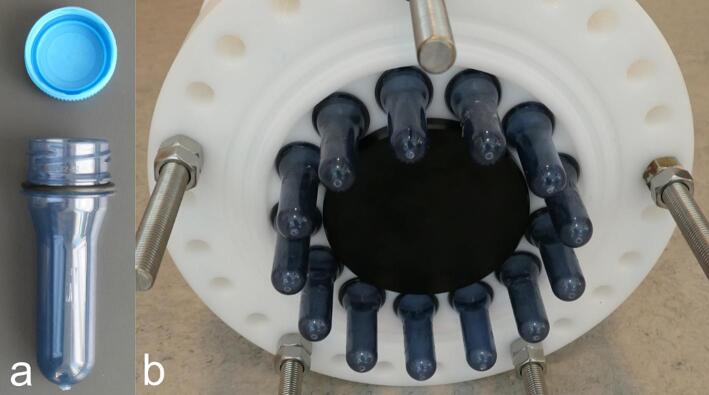
PET preform sample container with O-ring attached (a) and 13 sample containers attached to the valve manifold (b).

The sample containers for the instrument are 16 g polyethylene terephthalate plastic (PET) preforms with a 30/25H neck finish according to EN 16064:2011 [15]. A preform is injection molded drinks bottles before they are inflated to their final size. See Fig. 6a. This choice of component was made due to several factors:

1. They are cheap when bought in large quantities
2. Sturdy and able to hold the needed pressure for depths of 250 m
3. Clean as they are used in the food industry
4. Coarse thread for easy and quick installation and removal

Further, a large variety of preform weights and lengths are available with an identical thread [[16], [17], [18]]. This means that heavier preforms can be fitted to the instrument if greater sample depths or larger sample volumes are desired. The drawback of using preforms is that they can be difficult to source in smaller quantities.

**Fig. 7:**
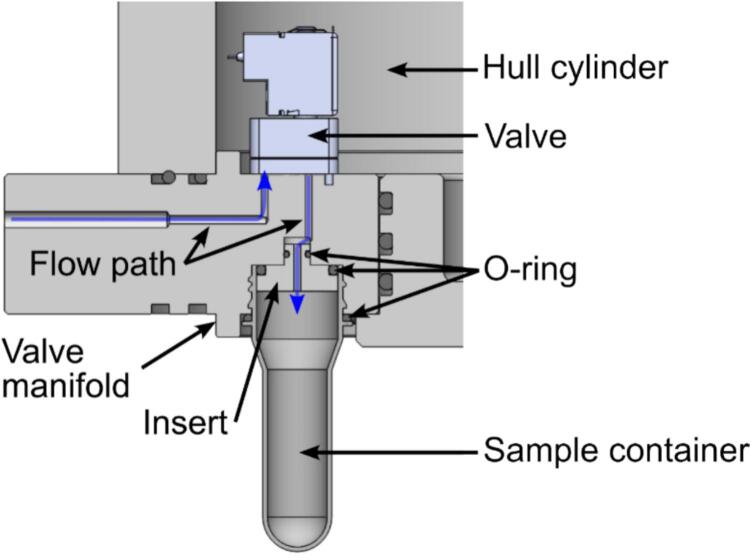
Section view of the valve manifold illustrating the flow path (blue) with the major components of the sampling subsystem annotated. (For interpretation of the references to color in this figure legend, the reader is referred to the web version of this article.)

The sample containers are fitted with an O-ring at the collar of the thread before they are attached to the hull using a threaded socket. See Fig. 6. A second O-ring seal is used on the inner face of the sample container via an insert in the valve manifold. This double O-ring seal ensures that no water can leak into the sample container, potentially contaminating the sample. See Fig. 7 for an overview of the water flow path, seals and insert locations.

### 2.3 Electronics

Four key design requirements for the electrical system were:

1. High level of user-friendliness and ease of use for efficient operation in small marine vessels (often in harsh weather conditions)
2. Simple assembly/disassembly
3. Profiling and mooring setup capabilities
4. Modular design allowing multiple valve manifolds to stacked together

The user interface consists of a single power switch and an LED indicator that signals the instrument operating mode mounted to the top of the hull. For charging, troubleshooting and configuring the instrument, an interface port is installed, through which the user can connect a PC to the instrument controller.

To allow multiple valve manifolds to be stacked and ease the assembly. The electronics are split into two printed circuit boards (PCB’s); a controller and a valve PCB. A single cable connects the controller PCB to the valve PCB, and additional valve PCB’s can be connected in a linear daisy chain topology.

The controller PCB houses the microcontroller that reads the attached pressure and temperature sensor and commands the valve PCB to actuate the valves. Logging information is saved to an on-board memory chip to allow verification of sample depths and timing. An on-board real-time clock is used to keep accurate timestamp information and periodically wake the instrument when operating in mooring mode. The valve controller houses the circuitry to power the valves on command from the controller. Power is supplied from a 14.4 V NiMH 3.8 AHr battery pack.

### 2.4 Software

**Fig. 8:**
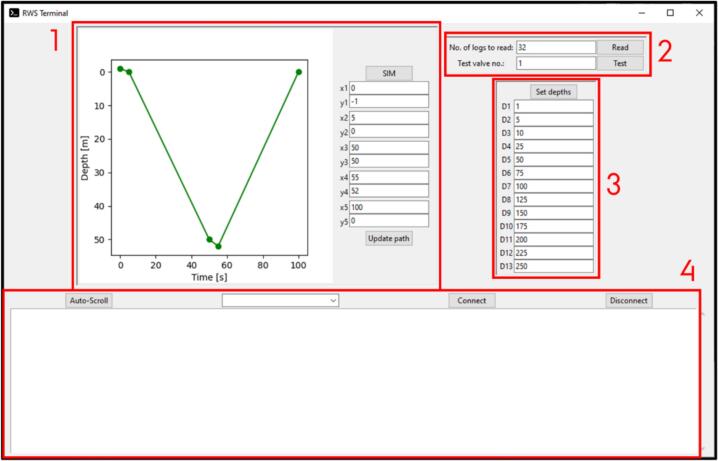
ARC-RWS Terminal user interface.

The instrument software is written in the C++ language using the Arduino framework [19] with the MiniCore core [20]. Communication with a PC for configuration and troubleshooting is accomplished via a serial interface. To simplify usage of the serial interface a Python program with a graphical user interface was developed. This allows the user to set the sample depths for all valves, read instrument log files, test individual valves and simulate a dive to verify correct operation. Further, debug messages from the instrument will be displayed. The interface is shown in Fig. 8 with sections marked:

1. Simulation panel used for testing instrument response
2. Log reading and valve testing
3. Set valve operating depths
4. Terminal window for connecting and reading incoming data stream

### 2.5 Price

**Fig. 9:**
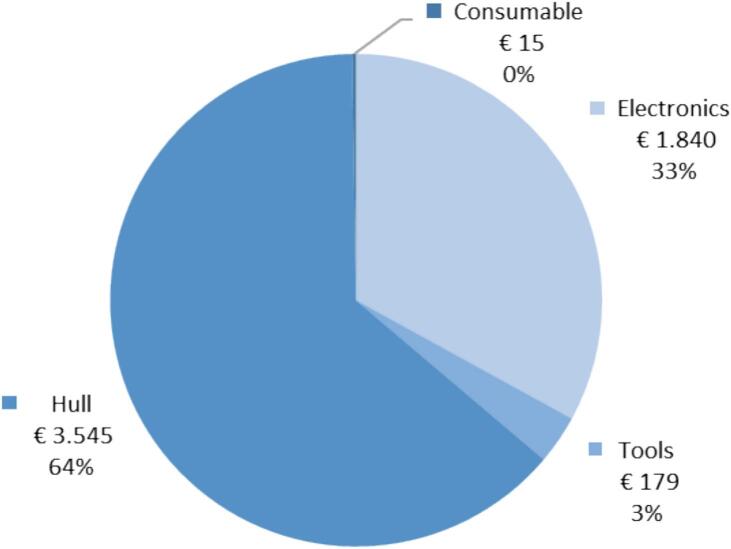
Cost breakdown for the ARC-RWS. Development and assembly cost not included.

Fig. 9 shows a breakdown of the cost of the ARC-RWS. The hull is the most expensive subsystem and amounts to 64 % of the total cost of € 5.578. This only includes the purchase cost of the components. The cost of development, assembly and testing is not included.

## 3 Design files summary

The main design files can be seen in Table 2, while a complete list of all design files needed for replicating the ARC-RWS can be seen in the repository located at https://doi.org/10.17632/b3jms757m9.2. Each directory in the repository contains a read-me file that provides further information about the design files in the directory.

**Table 2:** Summary of design files for the ARC-RWS. Files listed here are intended as a point of entry into the remaining design files.

Design file name	File type	Open source license	Location of the file
BOM.xlsx	Excel Workbook	CC BY 4.0	https://doi.org/10.17632/b3jms757m9.2
20003.sldasm	CAD file	CC BY 4.0	https://doi.org/10.17632/b3jms757m9.2
Controller_PCB.kicad_pro	KiCad Project	CC BY 4.0	https://doi.org/10.17632/b3jms757m9.2
Valve_PCB.kicad_pro	KiCad Project	CC BY 4.0	https://doi.org/10.17632/b3jms757m9.2
RWS_Software.sln	Visual Studio solution	CC BY 4.0	https://doi.org/10.17632/b3jms757m9.2
DS-Controller.ino	Arduino Sketch	CC BY 4.0	https://doi.org/10.17632/b3jms757m9.2
TS-Controller.ino	Arduino Sketch	CC BY 4.0	https://doi.org/10.17632/b3jms757m9.2
RWS Terminal.exe	Executable	CC BY 4.0	https://doi.org/10.17632/b3jms757m9.2
Log_interpreter.xlsx	Excel Workbook	CC BY 4.0	https://doi.org/10.17632/b3jms757m9.2

*BOM.xlsx* lists all parts needed to build the ARC-RWS and includes an index of all design files. Relationships between technical drawings and parts are defined in this file.

*20003.sldasm* is the SolidWorks 3D CAD assembly of the instrument. The assembly is also included as a STEP file for reading in other CAD software.

*Controller_PCB.kicad_pro* and *Valve_PCB.kicad_pro* are KiCad project files with documentation for PCB production, including schematics.

*RWS_Software.sln* is a Visual Studio Community with the Visual Micro plugin solution file. This links the different software libraries.

The firmware on the instrument is the Arduino Sketch *DS-Controller.ino* when operating as a Depth Sampler (profiling mode). When operating as a Time Sampler (mooring mode) the installed firmware should be the Arduino Sketch *TS-Controller.ino*.

*RWS Terminal.exe* is a GUI program to facilitate communications between the user PC and the instrument.

*Log_interpreter.xlsx* is an Excel spreadsheet to interpret the compressed data in the instrument log files. Data is parsed and presented in a human-readable format.

## 4 Bill of materials summary

A bill of materials for the ARC-RWS is included at the root of the linked repository with filename *BOM.xlsx*. A summary of the 15 most expensive components is listed in Table 3. The designator is the part number of the component, and references to this will be in parenthesis in the following sections.

**Table 3:** Summary of the complete BOM containing the 15 most expensive parts of the ARC-RWS. The complete BOM can be found in the supplementary design file BOM.xlsx.

Designator	Component	Number	Cost per unit − EUR	Total cost − EUR	Source of materials	Material type
10005-01-A	Valve manifold, plain	1	€ 1.125,55	€ 1.125,55	https://dk.nf-teknik.com/	Polymer
90002	Valve, X068544156001F3	13	€ 73,70	€ 958,06	https://www.emerson.com/	Metal
10009-A	Manifold vial insert	13	€ 65,86	€ 856,23	https://dk.nf-teknik.com/	Polymer
10004-01-A	Hull section cylinder single valve stack	1	€ 534,30	€ 534,30	https://dk.nf-teknik.com/	Polymer
10003-02-B	Interface lid	1	€ 467,64	€ 467,64	https://dk.nf-teknik.com/	Polymer
10001-01-B	Plain endcap	1	€ 412,03	€ 412,03	https://dk.nf-teknik.com/	Polymer
90009	SubConn Micro Circular Bulkhead connector 8 circuits	1	€ 199,52	€ 199,52	https://www.macartney.com/connectivity/subconn/subconn-micro-circular-series/subconn-micro-circular-2-3-4-5-6-and-8-contacts-and-g2-2-3-and-4-contacts/	Metal
10010-B	Controller PCB	1	€ 163,60	€ 163,60	https://aisler.net/	Semi-conductor
90003	Battery pack 14,4 V NiMH 3800 mAh (12S1P)	1	€ 136,58	€ 136,58	https://actec.dk/	Metal
10011-B	Valve PCB	1	€ 129,20	€ 129,20	https://aisler.net/	Semi-conductor
90004	Bar30 High-Resolution 300 m pressure Sensor	1	€ 76,56	€ 76,56	https://bluerobotics.com/store/sensors-cameras/sensors/bar30-sensor-r1/	Semi-conductor
90006	Celsius Fast-Response Temperature Sensor	1	€ 63,05	€ 63,05	https://bluerobotics.com/store/sensors-cameras/sensors/celsius-sensor-r1/	Semi-conductor
10008-01-A	M16 stainless threaded rod, 300 mm	5	€ 10,44	€ 52,19	https://ehandel.mw.dk/da/gevindstang-a2-og-a4/gevindstang-m16-a4	Metal
90030	SubConn Micro Circular inline cable, 8 circuits	1	€ 44,22	€ 44,22	https://www.macartney.com/connectivity/subconn/subconn-micro-circular-series/subconn-micro-circular-2-3-4-5-6-and-8-contacts-and-g2-2-3-and-4-contacts/	Polymer
00048	ISO 4032 − M16 − A4-70	20	€ 2,04	€ 40,73	https://ehandel.mw.dk/da/rustfri-staalmoetrik-din-934/moetrik-m16-a4	Metal

## 5 Build instructions

It is recommended to use design file *20003.sldasm* as a reference during assembly. General considerations to keep in mind during the entire assembly include:

- When handling watertight seals, it is essential to keep the seal clean and free of particles. Failure to do so can deteriorate the seal performance. Assemble the ARC-RWS in a clean environment.
- Scratches on sealing surfaces can cause leaks. Take care not to place any hull components directly on any faces with sealing surfaces.
- Cleaning components before assembly requires using isopropyl alcohol. Use appropriate safety equipment and procedures.

In addition to describing the construction of the instrument itself, the build instructions below also cover assembling the interface cable for communicating with the instrument and the pressure test tool to test the hull integrity.

### 5.1 Interface cable

The interface cable is used to charge and communicate with the instrument. To assemble it:

1. Strip the wire ends of the SubConn Micro Circular inline cable (90030)
2. Solder the wires to the pins of the USB to UART converter (90028) using Table 4 for reference. The silkscreen at the bottom of the converter labels each pin.

**Table 4:** Connection reference for the SubConn Micro Circular inline cable (90030) and the USB to UART converter (90028).

Cable color	USB to UART converter
White/Black	GND
Red/Black	VCC
Green	TX
Orange	RX
Blue	DTR
3. Install heat shrink tubing to the USB to UART converter (90028) to cover the exposed soldering joints.
4. Install heat shrink tubing and solder the red and black wire to the positive and negative terminal of the XT60 connector (90029) respectively. The connector has each terminal marked on the housing.
5. Connect the USB cable (90036) to the USB to UART converter (90028).

### 5.2 Pressure test tool

**Fig. 10:**
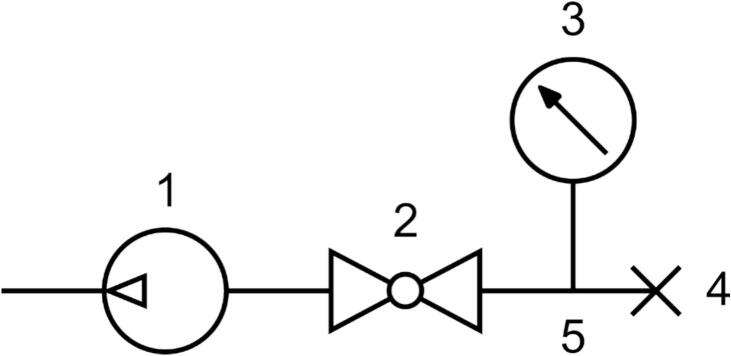
Schematic representation of the pressure test tool. (1) Vacuum pump, 90021, (2) ball valve, 90019, (3) vacuum manometer, 90024, (4) vacuum plug, 90026, and (5) tube Y connector, 90020.

The instrument is designed to be tested with a vacuum to verify the seals. To do this, assemble the pressure test tool:

1. Install a hose connector (90022) to the vacuum manometer (90024) using PTFE tape (90025) to seal the threads.
2. Install two hose connectors (90023) to the ball valve (90019) using PTFE tape (90025) to seal the threads.
3. Assemble the system as shown in Fig. 10, using polyurethane tubing (90027). Use a hot air gun to carefully heat the ends of the tubing to soften if necessary.

### 5.3 Top hull

Assembling the top hull section follows the procedure below:

1. Clean the O-rings and sealing surfaces for all sensors and penetrators (90004, 90005, 90006, 90007, 90008, 90009).
2. Lubricate the O-rings and install them on the penetrators.
3. Install the penetrators in the top hull (10003-02-B). It is recommended, but not required, to install as shown in design file *20003.sldasm*. The recommended installation was found to simplify the wiring.

### 5.4 Valve manifold

**Fig. 11:**
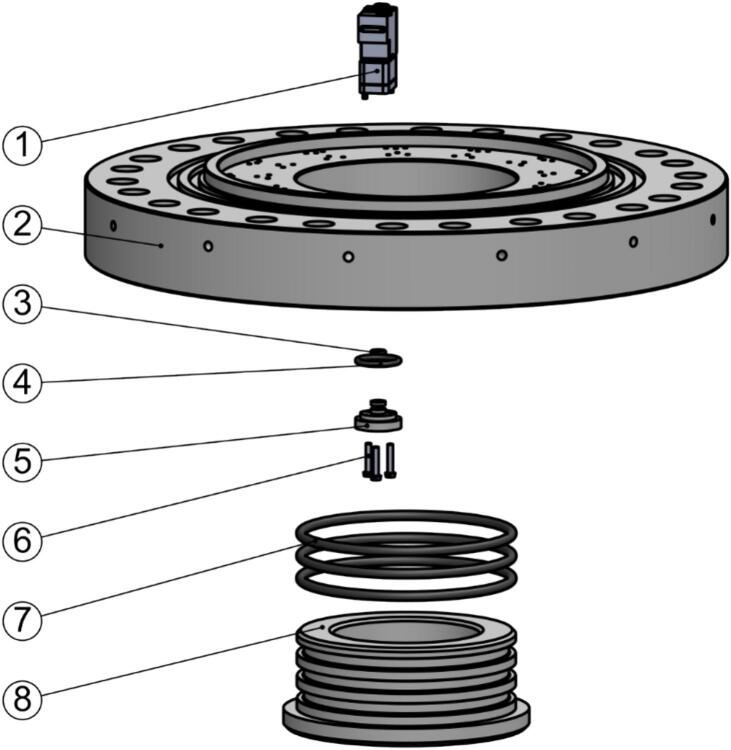
Exploded view of the valve manifold assembly. (1) Valve, (2) valve manifold, (3) O-ring 90012-05, (4) O-ring 90012-06, (5) insert, (6) ISO 1207-M3x16 nylon screw, (7) O-ring 90012-01, and (8) bottom endcap. Only a single valve and insert (along with accompanying O-rings and screws) are shown for simplicity.

Assembling the valve manifold is a three-step process. Use Fig. 11 as reference during assembly. First, install the sample container inserts:

1. Clean the O-rings (90012-05 and 90012-06) and sealing surfaces of the inserts (10009-A) and valve manifold (10005-01-A).
2. Lubricate the O-rings (90012-05 and 90012-06) with Molycote 111 (80003) and install them on the inserts (10009-A).
3. Push the inserts into the holes on the valve manifold (10005-01-A).
4. Secure each insert with three nylon screws (00056). Take care not to overtighten.

Second, install valves:

1. Inspect the gasket at the bottom of the valve (90002) for dirt or other contamination, and clean with isopropyl alcohol if necessary. Apply a thin layer of Molycote 111 (80003) to the gasket.
2. Clean the sealing surface on the valve manifold (10005-01-A) with isopropyl alcohol.
3. Install the valve with the included screws, making sure the inlet is furthest from the center (see design file *90002.jpg*).

Finally, install the bottom endcap:

1. Clean the O-rings (90012-01) and sealing surfaces of the endcap (10001-01-A).
2. Lubricate the O-rings (90012-01) with Molycote 111 (80003) and install them on the endcap (10001-01-A).
3. Clean the inner sealing surface of the valve manifold (10005-01-A).
4. Push the endcap (10001-01-A) into the valve manifold (10005-01-A) from the bottom, making sure it is fully inserted.

### 5.5 Electronics

The PCBs can be purchased already assembled and ready to install in the instrument. Alternatively, PCBs and components can be purchased separately for user assembly. This requires soldering skill and equipment but provides a lower purchase cost. In each case, the design files *Controller_PCB.kicad_pro* and *Valve_PCB.kicad_pro* contain the required specification for purchase and fabrication.

The controller PCB must be attached to the top hull assembly and soldered to the penetrators:

1. Mount the controller PCB (10010-B) to the center of the top hull assembly using double sided tape (90035). Make sure to clean the bonding surfaces with isopropyl alcohol for a proper bond. Orient the PCB so the cable connectors are accessible when the battery holder (10006-A) is installed.
2. Solder wires from the power switch (90007), indicator LED (90005), and interface port (90009) to the controller PCB (10010-B) according to Table 5.

**Table 5:** Connection reference for the controller PCB (10010-B) and indicator LED (90005), power switch (90007), and interface port (90009). PCB Connector column specify the name of the connector as indicated by the silkscreen on the PCB with the subfix specifying connector pin from left to right.

Part	Cable color	Signal	PCB Connector
Interface port	Black	Charge +	J2.2
	White	Not used	
	Red	Charge −	J2.1
	Green	TX	J3.4
	Orange	RX	J3.5
	Blue	DTR	J3.6
	White/black	GND	J3.1
	Red/black	3.3 V	J3.3
Power switch	Blue	Power +	SW1.1
	Blue	Power −	SW1.2
Indicator LED	Red	3.3 V	J10.1
	Black	GND	J10.2
3. Connect the temperature and pressure sensors (90006, 90004) to connector J8 and J9 on the PCB (10010-B), respectively.
4. Secure wires using hot glue to prevent strain on cable connectors and solder joints.
5. Solder jumper JP1 and JP3 pad 1 and 2 on the PCB (10010-B).
6. Install the real-time clock battery (80001) on the PCB (10010-B).
7. Install the battery holder (10006-A) in the top hull assembly using four M3x30 screws (00009).
8. Solder the connector cable (90033) to the battery (90003), using heat shrink tubing to cover the joint. Take care to avoid short circuiting the battery in the process. Ensure the polarity is correct.
9. Install double sided tape (90035) to the battery holder (10006-A) to prevent the battery from sliding.
10. Install the hook and loop strap (90013) in the battery holder (10006-A) and place the battery. Tighten the strap.
11. Insert the battery connector (step 4) into the connector at the top of the controller PCB (10010-B).

Installing the valve PCB to the valve manifold assembly is done as follows:

1. Mount the valve PCB (10011-B) to the center of the valve manifold assembly using double sided tape (90035). Make sure to clean the bonding surfaces with isopropyl alcohol for a proper bond. Orient the PCB so that the 13 LEDs on the board are directly in line with each valve (90002).
2. Solder wires from each valve (90002) to the pads next to it on the PCB (10011-B) and secure the wire with hot glue. Secure any access wire to the valves (90002) using a zip tie. See Fig. 12 for reference.

**Fig. 12:**
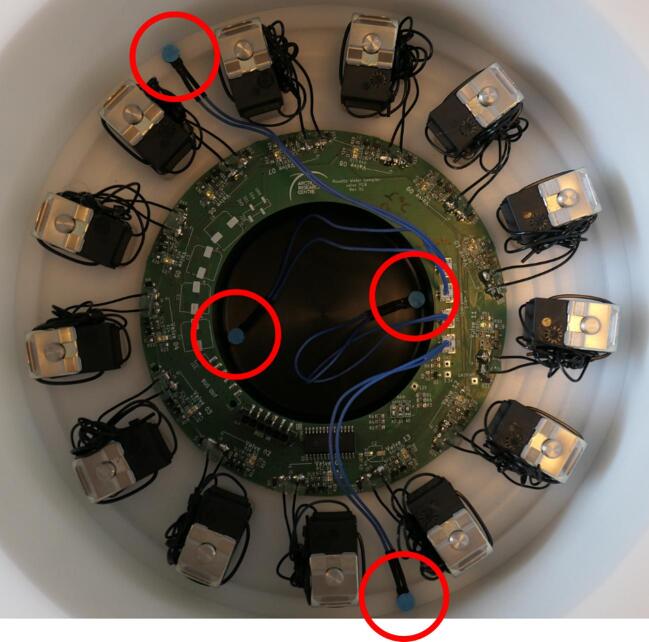
Valve PCB (10011-B) installed onto the valve manifold assembly. Leak probes (90015) and hull cylinder (10004-01-A) also installed. Leak probes marked with circles.
3. Solder jumpers A0-2 to configure the PCB I^2^C communication address. Setting all three to zero (soldering to minus) is the default.
4. Make sure to mark the valve numbering on the outside of the valve manifold (10005-01-A) for reference when using the instrument.
5. Install the leak probes (90015) as shown in Fig. 12 using the double-sided tape already attached to the probes.

To prepare the microcontroller on the controller PCB for programming:

1. Install the Arduino IDE [21], the MiniCore core [20], Microsoft Visual Studio [22] and Visual Micro [23].
2. Connect a 3.3 V Atmel AVR compatible in-system programmer (ISP) to the sockets at the bottom of the controller PCB (10010-B) according to Table 6. An Arduino can be used as an ISP. See [24] for reference.

**Table 6:** Connection table for programming the ARC-RWS bootloader using an in-system programmer.

Programmer	ARC-RWS Controller PCB
MISO	MISO_0
MOSI	MOSI_0
SCK	SCK_0
RST	Reset
VCC	3.3 V
GND	GND
3. Open *RWS_Software.sln* in Visual Studio.
4. Expand the “vMicro” toolbar and set the options specified in Table 7.

**Table 7:** Settings for uploading to the microcontroller on the controller PCB.

Parameter	Value
Board	ATmega328
Clock	Internal 8 MHz
BOD	BOD 2.7 V
EEPROM	EEPROM retained
Compiler LTO	LTO enabled
Variant	328 PB
Bootloader	Yes (UART0)
Baud rate	Default
5. Click “Uploader” and “Burn bootloader” on the”vMicro” toolbar.
6. Once successful, remove connections made in step 2.

The real-time clock on the controller PCB (10010-B) must be synchronized for proper timekeeping. To synchronize:

1. Connect the interface cable to the program port (90009) and to the user PC.
2. Open *RWS_Software.sln* in Visual Studio and open *DS3221_Set_Time.ino* from the Solution Explorer.
3. Expand the “vMicro” toolbar and set the options specified in Table 7.
4. Click “Build and Upload” on the toolbar.
5. Open a serial monitor and type the time to synchronize in the format “yy,mm,dd,hh,mm,ss” and hit enter.
6. This procedure will need to be completed each time the real-time clock battery is replaced.

To upload the firmware to the microcontroller:

1. Connect the interface cable to the program port (90009) and to the user PC.
2. Open *RWS_Software.sln* in Visual Studio and open *DS-Controller.ino* from the Solution Explorer.
3. Expand the “vMicro” toolbar and set the options specified in Table 7.
4. Click “Build and Upload” on the toolbar.

### 5.6 Testing

Testing should be completed before the instrument is fully assembled to allow easy access to components for troubleshooting. To test, insert the bus cable between the two PCBs (90018-02). Make sure to use the “BUS IN” connector on the valve PCB (10011-B). Then, follow the procedure below to connect to the instrument:

1. Turn off the instrument main power switch (90007).
2. Connect interface cable to both PC and instrument.
3. Open *RWS Terminal.exe*
4. Select the correct serial port in the drop-down menu.
5. Click “Connect”.
6. Wait 5–10 s.
7. Verify that the instrument is operating properly by inspecting the received data. If everything is working properly, it should be identical to the text below:

Booting up….

Setting state: 0.

Using depth table from EEPROM:

1, 5, 10, 15, 20, 25, 30, 50, 75, 100, 150, 200, 250.

Enabling I2C Bus.

Enabling RTC.

Enabling MEM.

Enabling TEMP.

Enabling BAR.

Enabling MCP.

Setting state: 1.

Writing to log… OK.

USB − Waiting for command….

Once connected, check that each valve is operating nominally:

8. Turn on the main instrument power switch (90007).
9. In the input field “Test valve no.:” input the valve number to test (i.e. 1-13) and click “Test”.
  a. Verify that the valve (90002) emits an audible click.
  b. Verify that the LED for the valve under test lights up.
  c. Verify flow through the valve by blowing compressed air into the sample inlet.

Still connected, check the instrument log to verify log operation and time synchronization:

1. Enter the number of log files to read from last to first (enter 0 to read all log files) in the “No. of logs to read” input in the top right. Click “Read”.
2. Insert the returned text into *Log_interpreter.xlsx* and verify results.

To power down and disconnect:

1. Turn off the main power switch (90007).
2. Disconnect the interface cable from the instrument and the user PC.

Once testing is completed remove the bus cable between the two PCBs (90018-02) in preparation for the main assembly.

Main assembly.

To finish the assembly, follow the procedure below:

1. Insert all five M16 threaded rods (10008-01-A) into the valve manifold (10005-01-A) with 120 mm protruding from the bottom. Use two M16 nuts (00048) on each threaded rod to set the height, and counter torque the two nuts to lock them in place.
2. Clean the O-rings (90012-02 and 90012-03) and sealing surfaces of the hull cylinder (10004-01-A) and valve manifold (10005-01-A).
3. Lubricate the O-rings (90012-02 and 90012-03) with Molycote 111 (80003) and install them on the valve manifold (10005-01-A) in the groves on the side with the valves installed.
4. Place the hull cylinder (10004-01-A) on the valve manifold.
5. If wanted, desiccant bags can be installed at this time.
6. Clean the O-rings (90012-02 and 90012-03) and sealing surfaces of the top of the hull cylinder (10004-01-A) and top hull (10002-02-B).
7. Lubricate the O-rings (90012-02 and 90012-03) with Molycote 111 (80003) and install them on the top hull (10002-02-B). This part must be installed with the O-rings suspended, so apply liberal amounts of grease at a few locations around the O-ring groves to help the O-rings stay in pace.
8. Insert the bus cable (90018-02) in the BUS IN connector on the valve PCB (10011-B)) and on the BUS OUT connector on the controller PCB (10010-B).
9. Taking care to prevent the O-rings falling out, gently lower the top hull assembly onto the rest of the instrument using the threaded rods as a guide. Check that no wires are getting in between the top hull (10003-02-B) and the hull cylinder (10004-01-A).
10. Visually verify that the O-rings have not shifted during assembly.
11. Install five M16 nuts (00048) on the top hull assembly and tighten until no gap is visible on either side of the hull cylinder.
12. Lubricate the interface port with Molycote 44 (80002) and install the dummy plug (90010) and locking sleeve (90011).
13. Attach rope through the external holes of the instrument to enable attachment to a winch line and ballast weights. See Fig. 2a.

The instrument is now fully assembled. Pressure testing should be carried out to find any leaks before deployment:

1. Unscrew the valve port cap (90008) and insert the plug of the pressure test tool into the port.
2. Apply 12 V DC to the vacuum pump (90021) and open the ball valve (90019).
3. Wait for the pressure indication on the manometer (90024) to settle before closing the valve (90019) and turning off the pump (90021).
4. Note the pressure indication on the manometer and wait for a minimum of one hour.
5. Note the pressure indication on the manometer again and compare with the previous pressure measurement. If no pressure difference is observed, the instrument has passed the test.

## 6 Operation instructions

Operating the ARC-RWS is completed on boats or near bodies of water. Use appropriate safety equipment and procedures to prevent any accidents related to water. Further, the battery voltage of the instrument is 16.8 V maximum. In dry conditions, this is well below what is generally considered potentially harmful voltage levels (>30 V). However, in wet conditions, like when operating the instrument, low voltages can, on very rare occasions, be harmful. If the instrument is behaving unexpectedly, use dry rubber gloves to handle the instrument or make sure you are completely isolated from any potential current paths to prevent shocks.

Prior to deployment the battery must be charged. To charge the instrument, do as follows:

1. Turn off the main power switch (90007) on the instrument.
2. Connect the XT60 connector (90029) of the interface cable and power on the charger (90031).
3. Set charging current to 0.30 A and NiMH battery chemistry.
4. Connect the interface cable to the interface port (90009).
5. Press and hold ENTER to start charging.
6. Monitor the charging process and terminate if any of the below conditions are met:
  a. Charging exceeds 8 h.
  b. Voltage exceeds 17 V.
  c. Voltage stays constant (± 0,05 V) for more than 1 h.
7. Press STOP to stop charging.
8. Disconnect interface cable from charger and instrument.

Monitor the battery voltage regularly, and recharge when voltage is 14.4 V or less. To configure the sample depths, connect to the instrument as described in section 5. In the right side of the screen, set each sample depth and click “Set depths”. The instrument will confirm the operation in the output window.

The instrument is now ready for deployment:

1. Install O-rings (90012-04) on 13 sample containers (90001) and screw into the bottom of the instrument. Use a pipe wrench to tighten the sample container.
2. Turn on the main power switch (90007) and verify that the LED (90005) blinks two times each second. A rapid continuous blinking indicates an error. In this case, connect to the instrument and diagnose immediately using the reported error code in conjunction with Table 8.

**Table 8:** Error bitmask and resolution.

Error	Bitmask	Corrective measure
I2C communication	1	Check wiring from valve PCB to controller PCB
Real-time clock	2	Check real-time clock battery (80001) voltage (>2.3 V) and replace as needed
Log memory	4	Reboot instrument. Replace controller PCB if error persists
Temperature sensor	8	Check for sensor damage and check wiring to controller PCB
Pressure sensor	16	Check for sensor damage and check wiring to controller PCB
Valve port	32	Check wiring from controller PCB to valve PCB
Leak	64	Investigate the source of the leak. Clean and lubricate the compromised seal, or replace components as needed
Valve depth table	128	Reboot instrument. If error persists, reset valve depths
3. Lower the instrument to the water surface. If sample depths have been set close to the surface (<5 m) lower the instrument very slowly (< 0.25 m/s). At shallow depths the pressure difference between the water and sample volume is not great enough to fill the sample rapidly.
4. Resume profiling at desired velocity (max 1 m/s).
5. Once resurfaced, check the LED (90005): A rapid continuous blinking indicates an error. In this case, connect to the instrument and diagnose immediately using the reported error code in conjunction with Table 8.
6. Turn off the main power switch (90007).
7. While keeping the instrument level, remove the sample containers (90001) and install the lid to secure the sample.
8. If the instrument will not be deployed again immediately, rinse with freshwater and allow it to dry.
9. To verify correct sample depths the instrument log files can be downloaded and parsed as described in section 5.

If one or more of the sample containers are not filled upon resurfacing the most likely issue is a clogged valve. This can usually be fixed by the following:

1. Connect to the instrument as described in section 5.
2. Set the clogged valves to a depth greater than 200 m.
3. Install sample containers and perform a profiling operation as previously described.

The high pressure has been found to effectively clear the water flow path through the instrument and valve.

Instrument errors are reported as an 8-bit error bitmask. Each bit represents a distinct error. Refer to Table 8 for a description of each error and what corrective measures need to be taken.

## 7 Validation and characterization

**Fig. 13:**
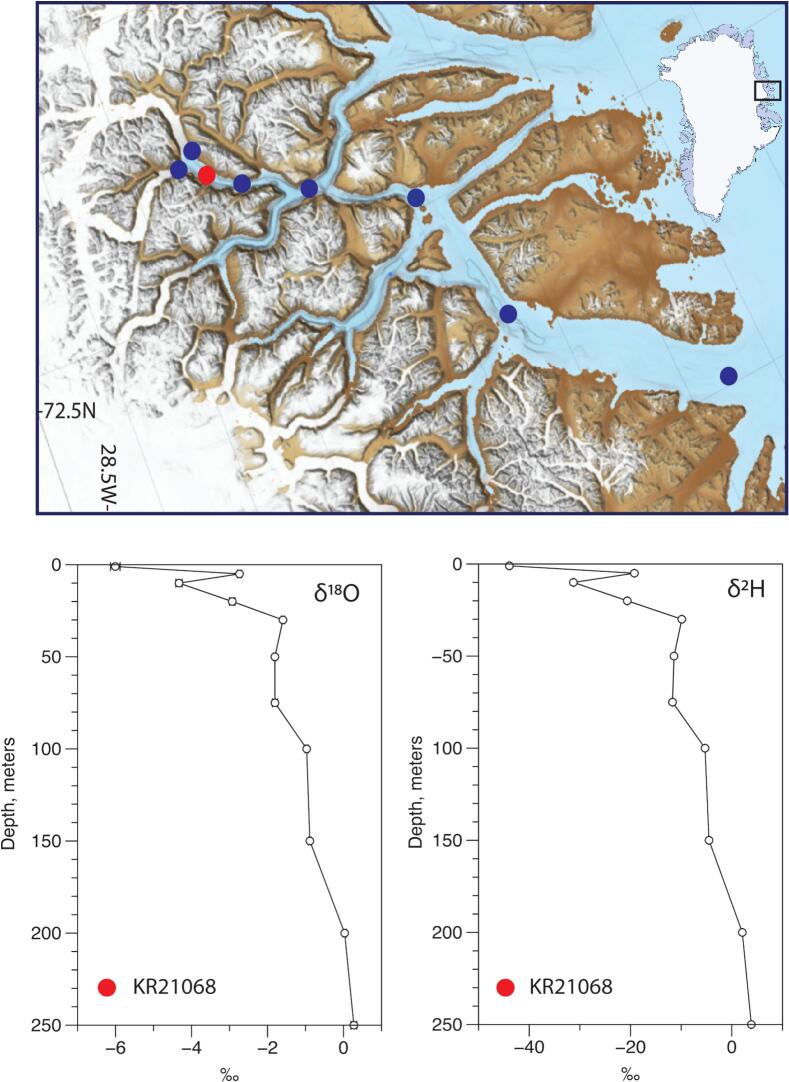
Water sample stations along the Isfjord-Kong Oscar Fjord section in Northeast Greenland (upper panel). Data example of water isotopes from station KR21068 (lower panel). Error bars (SE) are calculated based on 3 replicate samples.

The ARC-RWS have been used during three field campaigns in Northeast Greenland in the Ella Ø area (see Fig. 13) and along the East Greenland coast between 2021 and 2023. Here, the instrument has been tested to depths of 250 m using both manual winches on small two-person boats and automatic winch systems on large crane-equipped marine vessels. In total more than 25 profiling casts have been completed without damage or failure. Further, the instrument has been installed successfully three times in a mooring configuration at a depth of 8 m for a full year.

Testing the sample container integrity has been carried out to depths of 250 m. The transparent sample containers were visually inspected post-profiling to verify that no water entered.

Water samples for stable hydrogen (δ^2^H) and oxygen isotope (δ^18^O) analysis were collected using the ARC-RWS instrument at various locations along the Isfjord and Kong Oscar Fjord, ranging from the glacier front to the coast in the eastern Greenland Sea (Fig. 13). Sampling was conducted at depths of 1, 5, 10, 20, 30, 50, 75, 100, 150, 200, 210, 220, and 250 m. The water samples were transferred to 2 mL glass vials and stored at room temperature until analysis.

The δ^2^H and δ^18^O concentrations were determined using a Cavity Ringdown Spectrometer (L2130-i Isotopic H_2_O Analyzer, Picarro Inc., USA). These stable isotope analyses helped trace the origin of freshwater within the fjord system. In Greenland, δ^18^O values in glacial water are typically –27.7 ± 0.2 ‰, while Atlantic Water and Polar Water exhibit values of 0.23 ± 0.03 ‰ and −1.20 ± 0.10 ‰, respectively [25]. The depleted δ^2^H and δ^18^O isotopic signatures observed in the upper 100 m indicate that glacial meltwater from the Greenland ice sheet has mixed into this layer.

Further details on the methods and results obtained with the ARC-RWS instrument can be found in [26].

During fieldwork, the instrument was operated reliably by a single person. With proper sample container preparation (O-ring installation and numbering) the time to prepare the instrument between sampling was found to be about 10 min.

### 7.1 Improvements

While the ARC-RWS has demonstrated that it fulfills the requirements listed in Table 1, some areas have been identified for further improvement. This is mainly to address ease of use and assembly.

*Assembly*.

When closing the hull by placing the top hull on the hull cylinder the two large O-rings of the top hull can easily fall out. This makes assembling the instrument in a controlled manner significantly more difficult. Placing the O-ring groves on the hull cylinder will fix this problem.

During use the leak probes have been shown to often come loose. This decreases the leak detection reliability. A mechanical mounting method or different bonding technique must be considered.

*Design*.

A failure of a valve seal will in the present state allow water to enter the main hull. As there are 13 valves, in addition to the other hull seals, this greatly increases the risk of failure. This failure has been observed once after operation. A design that is tolerant to valve seal failures should be investigated.

To improve the handling experience of the instrument, especially in small marine vessels, a weight reduction would be needed. The current weight can be reduced by making the instrument smaller. The size is, in large, driven by the size of the valves and sample containers. This means that to effectively reduce the size the valves and sample containers must be placed in a different configuration or reduced considerably in size. Improvements could include replacing the plastic hull with aluminum and changing the current axial valve and sample container pattern to a radial pattern. This will allow for multiple rows of valves and sample containers, thus reducing the hull diameter significantly.

In relation to this, the sample tubes can be difficult to install and remove due to the tight spacing between each sample container. Improving this will dramatically improve the user experience.

The large flat disc shape of the instrument creates significant drag during downcast. This can be alleviated by increasing the ballast weight, at the cost of user handling. Adding a cowling to the instrument is thought to be able to reduce the drag.

Finally, adding handles to the instrument would ease handling.

*User interface*.

The user interface has been shown to be intuitive and efficient when operating the instrument. However, when an error is encountered, the instrument firmware could be improved to more effectively assist the user in diagnosing and fixing the error.

Further, connecting to the instrument requires a specific procedure. This can be simplified with more intelligent firmware on the instrument that is more tolerant to the procedure used when connecting.

## Ethics statements

Not relevant.

## CRediT authorship contribution statement

**Ebbe Poulsen:** Writing – original draft, Validation, Software, Project administration, Investigation. **Søren Rysgaard:** Writing – review & editing, Validation, Supervision, Investigation, Funding acquisition, Formal analysis, Conceptualization. **Peter Melvad:** Validation, Software, Conceptualization. **Claus Melvad:** Writing – review & editing, Supervision, Conceptualization.

## Declaration of competing interest

The authors declare that they have no known competing financial interests or personal relationships that could have appeared to influence the work reported in this paper.
